# Variation of nanoparticle fraction and compositions in two-stage double peaks aging precipitation of Al−Zn−Mg alloy

**DOI:** 10.1186/s11671-018-2542-1

**Published:** 2018-04-27

**Authors:** Y. L. Wang, Y. Y. Song, H. C. Jiang, Z. M. Li, D. Zhang, L. J. Rong

**Affiliations:** 1 CAS Key Laboratory of Nuclear Materials and Safety Assessment, Institute of Metal Research, Chinese Academy of Science, Shenyang, 110016 China; 2 School of Materials Science and Engineering, University of Science and Technology of China, Hefei, 230026 China

**Keywords:** Atom probe tomography (APT), Al−Zn−Mg alloy, Two-stage double peaks aging, Fraction, Compositions

## Abstract

Atom probe tomography (APT) coupling high-resolution transmission electron microscopy (HRTEM) was used to analyze the fraction and compositions of different nanoparticles in two-stage double peaks aging process of Al−Zn−Mg alloy. Al content is found to be closely related to the size of nanoparticles and it can be greater than ~ 50.0 at. % in the nanoparticle with the equivalent radius under ~ 3.0 nm. Correspondingly, Al content of the nanoparticle, with the equivalent radius over ~ 5.0 nm, is measured under ~ 40.0 at. %. Evolution from Guinier–Preston (G.P.) zone to η phase is a growing process where Mg and Zn atoms enter the nanoparticle, therefore rejecting Al atoms. G.P. zones can take up a number fraction of ~ 85.0 and ~ 22.7% of nanoparticles in the first and second peak-aged samples, respectively, and even in the over-aged (T73) sample, they can still be found. As aging time increases, fraction of η′ phases monotonically rises to the peak value (~ 54.5%) in the second peak-aged state and then drops, which is significant for the second hardness peak and directly proves their function as the transitional medium. In T73 state, ~ 63.3% nanoparticles compose of η phases, which were measured to still contain ~ 10.2 to ~ 36.4 at. % Al atoms.

## Background

Aging treatment is an indispensable way to strengthen Al−Zn−Mg−(Cu) alloys [1–3]. In the last century, a primary agreement on precipitation sequence of Al−Zn−Mg alloys has been reached: Supersaturated Solid Solution → Coherent Guinier–Preston (G.P.) zones→ Semi-coherent intermediate η′ phases→ Incoherent equilibrium η (MgZn_2_) phases [4]. Previous works have found double hardness peaks in the two-stage aging process of Al−Zn−Mg alloys and put forward that the two hardness peaks were mainly contributed by G.P. zones and η′ phases, respectively [5, 6]. The strengthening effect of G.P. zones and η′ phases is much stronger than that of η phases [7], and we found that matrix precipitates (MPts) in each state of aging process are not single in type, so that the fractional variation of each kind of nanoparticles can further affect mechanical properties of Al−Zn−Mg alloys. However, the fraction of those nanoparticles in different aging states is difficult to be analyzed only by transmission electron microscopy (TEM) due to the limitation of two-dimensional observation. Meanwhile, the compositions of nanoparticles is another significant parameter, which can further influence the property such as corrosion resistance of Al−Zn−Mg alloys [8]. However, energy-dispersive spectroscopy (EDS) cannot accurately measure the compositions of nanoparticles. Atom probe tomography (APT), a novel alternative high-resolution characterization method providing three-dimensional (3D) elemental information, can precisely measure both the compositions and fraction of nanoparticles. Some works through APT have focused on the compositions of nanoparticles in aged Al−Zn−Mg alloys, but the results are multifarious about the Zn/Mg ratio and Al content [9–15]. At the same time, researchers have not focused on the fraction of different nanoparticles in the whole aging process by performing APT analysis. In this work, we combine APT with high-resolution transmission electron microscopy (HRTEM) to reveal the variation of nanoparticle fraction and compositions in Al−Zn−Mg alloy and are aimed at providing guidance for better choice of aging regime.

## Methods

### Material

A medium-strength Al−Zn−Mg alloy (7N01) was used in the current study. The chemical compositions are given as follows (in wt.%): 4.06 Zn, 1.30 Mg, 0.30 Mn, 0.18 Cr, 0.13 Zr, 0.05 Ti, and balance Al. The extruded alloy was quenched with water spray at room temperature, followed by 72-h natural aging and then treated by two-stage artificial aging.

### Characterization

Hardness test were carried on the microhardness tester to characterize the aging-hardening behavior. HRTEM was performed on FEITecnai F20 to identify the precipitates. The APT characterization was conducted on a CAMECA Instruments LEAP 5000 XR local electrode atom probe with energy-compensation reflectron. Specimens for atom probe were prepared by a two-step electro-polishing procedure. The first step used an electrolyte of 10% perchloric acid in acetic acid, and the second step used an electrolyte of 4% perchloric acid in 2-butoxyethanol. The APT test was performed at 50 K with the voltage pulsing rate at 200 kHz. Imago Visualization and Analysis Software (IVAS) version 3.8.0 was used for 3D reconstructions and composition analysis. 12.0 at. % (Mg+Zn) isoconcentration surface was applied to visualize the nanoparticles including G.P. zones, η′ phases, and η phases.

## Results and Discussion

**Fig. 1:**
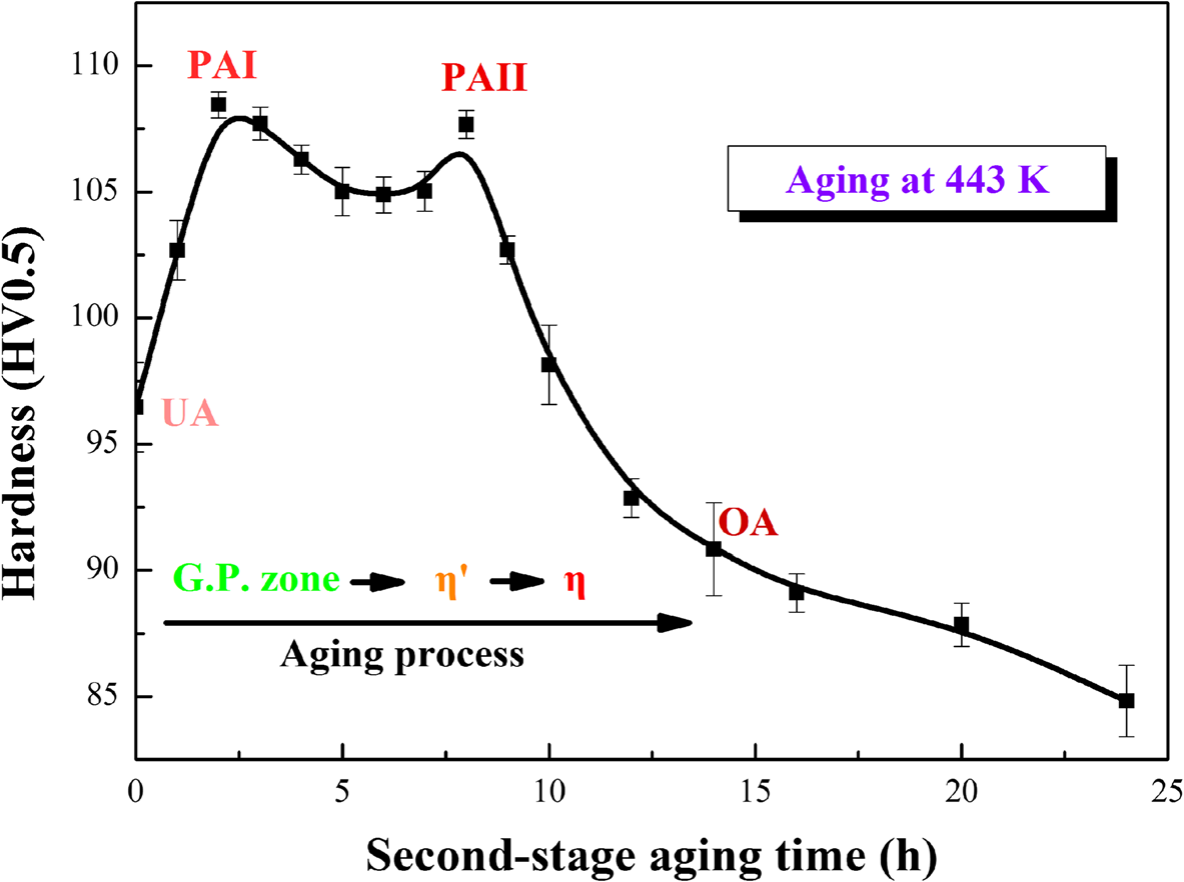
Aging-hardening curve of the experimental alloy in the second-stage aging process

The experimental alloy was subjected to a two-staged aging treatment, i.e., aged at 373 K for 12 h and then aged at 443 K for different times. The second-stage aging-hardening curve of experimental alloy is shown in Fig. 1. The states at 0, 2, 8, and 14 h of the second-stage aging process were corresponded to UA (under aging), PAI (peak aging I), PAII (peak aging II), and OA (T73 in over aging), respectively. According to hardness variation, alloy in T73 state loses ~ 15% hardness compared to PAI.

**Fig. 2:**
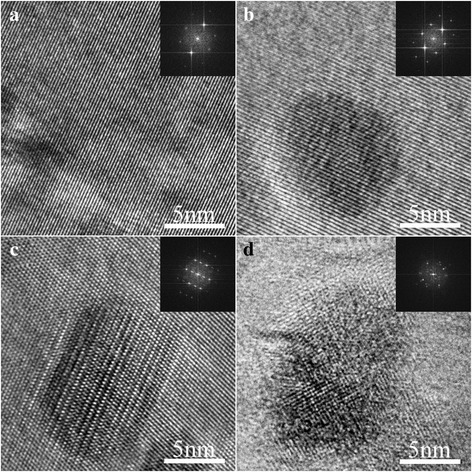
BF HRTEM images of typical nanoparticles in different states of the second-stage aging process: **a** UA, **b** PAI, **c** PAII, and **d** OA. Selected area electron diffraction (SAED) patterns near [110], [011], [011], and [001] zone axis are shown as insets in **a**–**d**, respectively

Typical nanoparticles in such four states were observed by HRTEM, and the bright field (BF) images are shown in Fig. 2. The fully coherent relationship between the nanoparticle and Al matrix near [110] zone axis in Fig. 2a directly proves the presence of G.P. zone in UA [16]. As aging time lengthens, G.P. zone coarsens in PAI and is still coherent with Al matrix as shown in Fig. 2b. For the nanoparticle shown in Fig. 2c, the lattice distortion can be clearly seen, which is related to the procedure that Zn atom moves into the lattice and induces disorder in the η′ phase [17]. Meanwhile, the previous research also reported that the second aging peak is mainly caused by the η′ phase [6]. However, the typical nanoparticle in OA exhibited in Fig. 2d is totally incoherent with Al matrix and shows hexagonal lattice near the [001] zone axis, which can be recognized as η phase. Specifically, the *a* axis is measured at ~ 0.53 nm and agrees well with the previous study on the equilibrium η phase [18].

**Fig. 3:**
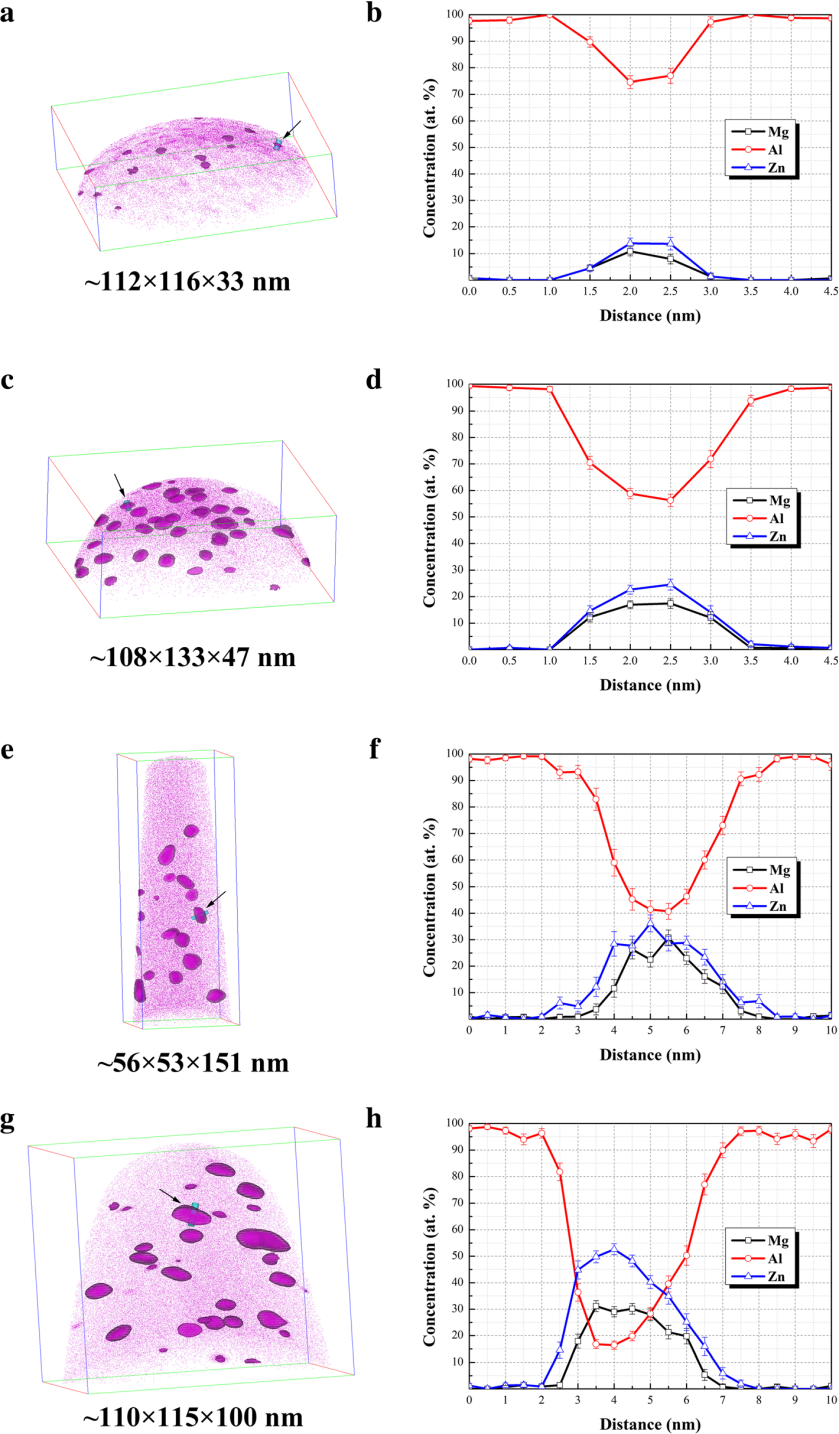
Three-dimensional reconstruction of specimens in different second-stage aging states: **a** UA, **c** PAI, **e** PAII, and **g** OA. The composition profiles through marked typical nanoparticles in **a**, **c**, **e**, and **g** were measured using a selected cylinder (diameter, 3 nm) with a moving step of 0.5 nm and shown in **b**, **d**, **f**, and **h**, respectively

**Fig. 4:**
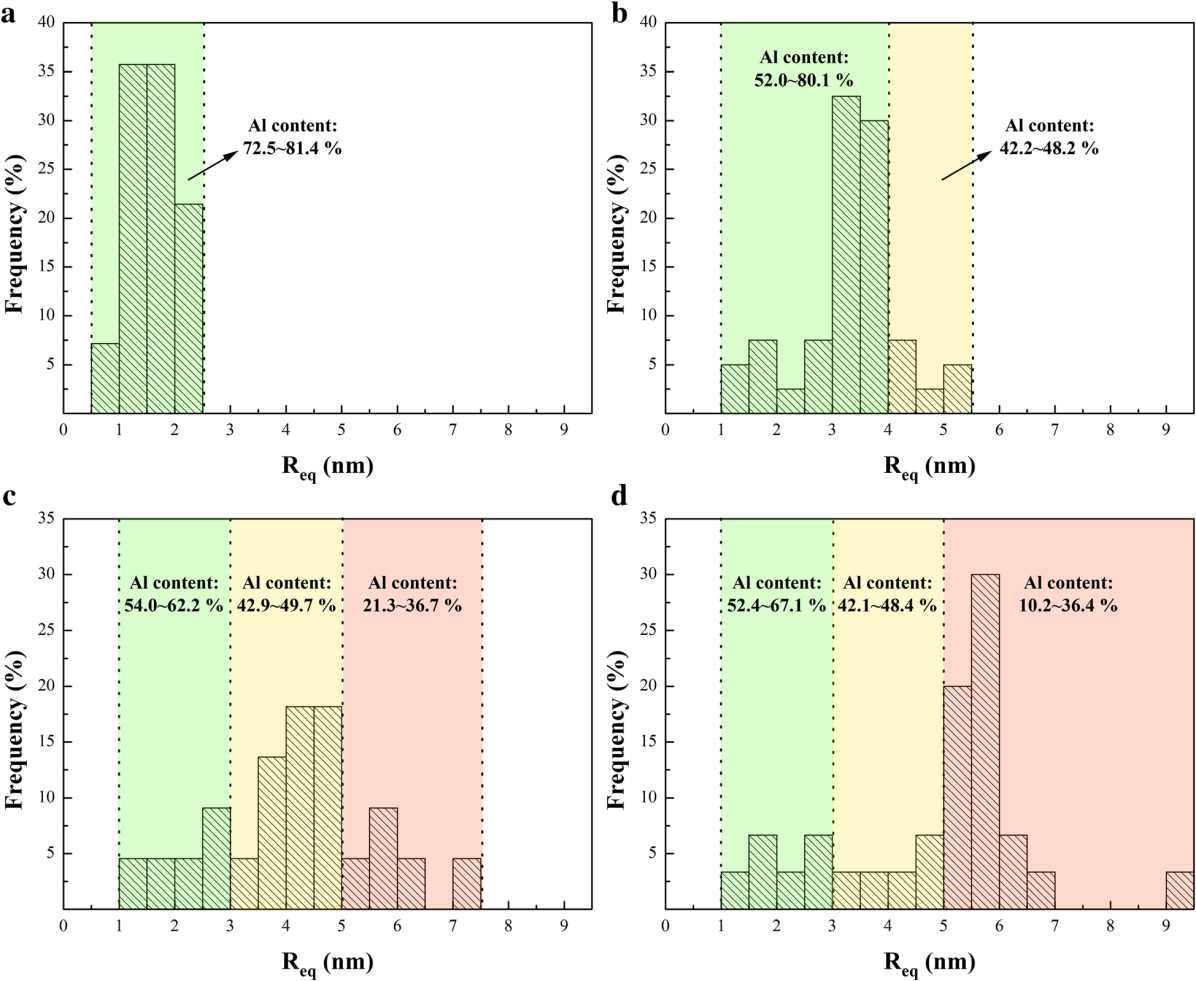
Distribution of equivalent radius (*R*_eq_) and the corresponding Al content (in at. %) of the nanoparticles in different second-stage aging states: **a** UA, **b** PAI, **c** PAII, and **d** OA

**Fig. 5:**
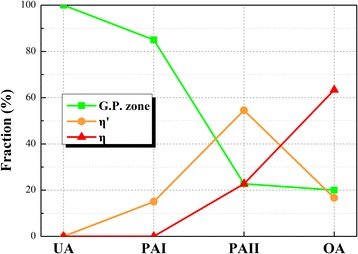
Statistical fraction of nanoparticles in different second-stage aging states

Figure 3 demonstrates the 3D reconstruction morphology of specimens in different second-stage aging states, together with representative 1D concentration profiles through the marked typical nanoparticles in each states. The image shown in Fig. 3a represents the nanoparticles in early stage aging that consist of G.P. zones. As shown, relatively small amount of tiny nanoparticles can be observed. The concentration analysis shown in Fig. 3b indicates that the typical nanoparticle with ~ 2.0 nm in thickness varies in compositions with an average content of ~ 13.8 ± 0.1 at. % Zn, ~ 9.4 ± 2.1 at. % Mg and ~ 75.8 ± 1.7 at. % Al, and a Zn/Mg ratio at ~ 1.5: 1. The hardness peak in PAI is mainly contributed by G.P. zones [6]. In the reconstruction morphology of specimen in PAI (Fig. 3c), a great deal of flat nanoparticles can be clearly observed. The average compositions of the typical nanoparticle in Fig. 3c were measured as ~ 23.6 ± 1.3 at. % Zn, ~ 17.2 ± 0.3 at. % Mg, and ~ 57.5 ± 1.8 at. % Al, giving an average Zn/Mg ratio at ~ 1.4: 1, and the thickness rises to ~ 2.5 nm as demonstrated in Fig. 3d. The compositions of above-mentioned nanoparticles in UA and PAI state are both consistent with the previous result about G.P. zone of which Zn/Mg ratio was found to lie between 1:1 and 1.5:1 [9, 10, 12]. Figure 3e exhibits the reconstruction morphology for specimen in PAII state, of which corresponding HRTEM results indicate that the major nanoparticles are the η′ phases. It can be clearly seen that the nanoparticles tend to be ellipsoidal in shape. Meanwhile, compared to G.P. zones, a mass of Al in the typical nanoparticle was replaced by Zn and Mg solutes as shown in Fig. 3f. Specifically, there are about ~ 30.3 ± 3.9 at. % Zn and ~ 25.7 ± 3.8 at. % Mg together with ~ 43.4 ± 2.8 at. % Al within the nanoparticle, and the average Zn/Mg ratio is measured at ~ 1.2:1. As shown in Fig. 3g, it is coincident with the HRTEM observation that most typical nanoparticles in OA coarsen in size. Corresponding to the hardness decline during over aging, the η phase shows quite weak strengthening effects on experimental alloy. In detail, the ~ 6.0-nm-thick typical nanoparticle mainly consists of ~ 50.2 ± 2.2 at. % Zn and ~ 30.1 ± 1.1 at. % Mg together with ~ 17.7 ± 1.9 at. % Al and possesses a Zn/Mg ratio of approximately ~ 1.7:1. Meanwhile, we found that the equivalent radius (*R*_eq_) of nanoparticles is related to the Al content. Figure 4 shows the distribution of *R*_eq_ and the corresponding Al content of nanoparticles in different aging states through statistical analysis of over hundred nanoparticles. It can be easily found that the larger the particle, the less Al it contains. For explanation, evolution from G.P. zone to η phase is a growing process where Mg and Zn atoms enter the nanoparticle, therefore rejecting Al atoms. Firstly, we found that the Al content of nanoparticles in OA can be divided into three ranges with the *R*_eq_ increases, as shown in Fig. 4d. In detail, when *R*_eq_ is beyond ~ 5.0 nm, the Al content ranges from ~ 10.2 to ~ 36.4 at. %. Such composition is similar with the chemical study of the η phase reported by Maloney [14]. Correspondingly, it varies from ~ 42.1 to ~ 48.4 at. % and from ~ 52.4 to ~ 67.1 at. % when *R*_eq_ is between ~ 3.0 and ~ 5.0 nm and lower than ~ 3.0 nm, respectively. More interestingly, PAII condition in Fig. 4c shows a similar result. Therefore, by referencing the present and previous APT results [9, 14], we divide the Al content into three ranges, i.e., > ~ 50.0, ~ 40.0 to ~ 50.0, and < ~ 40.0 at. %, and correspondingly divide *R*_eq_ into three ranges, i.e., < ~ 3.0, ~ 3.0 to ~ 5.0, and >~ 5.0 nm, so as to distinguish the G.P. zones, η′ phases, and η phases. Undoubtedly, the nanoparticles in UA (Fig. 4a) with ~ 72.5 to ~ 81.4 at. % Al are totally G.P. zones. However, Fig. 4b shows that *R*_eq_ of nanoparticles in the PAI can reach ~ 4.0 nm though the Al content still beyond ~ 50.0 at. %. Those relatively coarse G.P. zones may be the precursors of η′ phase of which size exceeds the critical size and they can partly lose coherent relationship with Al matrix. As a result, the relationship between nanoparticles constitution and aging time can be revealed. Figure 5 shows the statistical fraction of nanoparticles in different aging states. G.P. zones take up ~ 85.0 and ~ 22.7% nanoparticles in the first and second peak aging alloy. As aging time increases, fraction of G.P. zones decreases and that of η′ phases monotonically rises to the peak value (~ 54.5%) in PAII and then drops, which directly proves their function as the transitional medium. After T73 aging treatment, there are ~ 63.3% η phases in the OA and G.P. zones still taking up ~ 20.0% of the nanoparticles. Therefore, the double hardness peaks are both contributed by G.P. zones and η′ phases. G.P. zones take up the main hardening nanoparticles in the first peak-aged alloy, while most of them transfer to η′ phases in the second peak-aged alloy and then η′ phases becomes the major hardening phases. Furthermore, decrease of hardness in OA is directly related to the formation of η phases which show weaker hardening effect than G.P. zones and η′ phases [7].

**Fig. 6:**
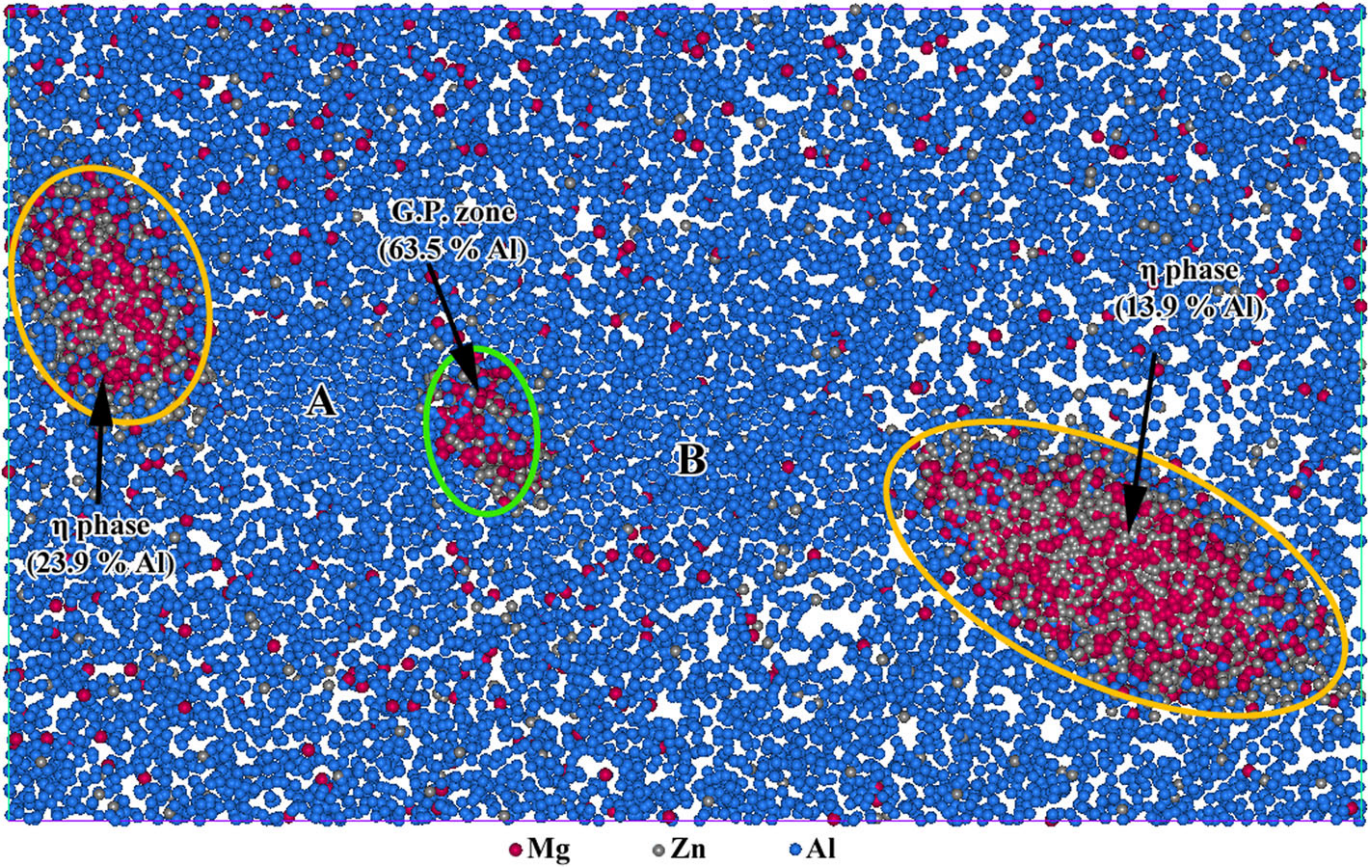
Typical 1-nm-thick atom map (50 × 30 nm) showing the distribution of Mg, Zn, and Al atoms in OA state. The corresponding Al content within nanoparticles were shown as inset

As mentioned, a certain quantity of G.P. zones still exists after sufficient aging. Figure 6 shows the typical atom map in OA state, in which the coexistence of G.P. zone and η phase can be clearly observed. η phases are marked in yellow, while G.P. zone is in green. Interestingly, regions marked in A and B between the G.P. zone and η phases are comparatively Al rich and Mg and Zn poor than other regions. It is believed that from the beginning of the aging treatment, nanoparticles at the two sides can grow faster than the one between them. As a result, such two relatively large nanoparticles are easy to capture when surrounding Mg and Zn atoms in the marked A and B areas and can further transform to precipitates, which directly restrict the growth of the G.P. zone between them. Therefore, the G.P. zone grows quite slow and can exist after sufficient aging treatment. Moreover, it also can be a dissolving process of such G.P. zone by transferring Mg and Zn atoms to the two larger η phases in case of its size is lower than the critical one.

## Conclusions

1. Nanoparticles in the first peak-aged Al−Zn−Mg alloy consist of ~ 92.5% G.P. zones, of which Al content are all beyond ~ 50.0 at. %. The highest hardness value corresponding to the first peak-aged state is mainly contributed by G.P. zones.
2. The second hardness peak is contributed by both η′ phases and G.P. zones, which take up ~ 54.5 and ~ 22.7% of the nanoparticles, respectively. Al content of intermediate η′ phases lies between that of G.P. zones and η phases.
3. Al content in the η phase is found to be lower than ~ 40.0 at. % and their equivalent radius are larger than ~ 5.0 nm. No η phase forms in the under-aged and the first peak-aged aging state, while it takes up ~ 63.3% of the nanoparticles in the T73 state. Those η phases in T73 state still contains ~ 10.2 to ~ 32.4 at. % Al, which can further decrease with the extension of aging time.
4. Growth of G.P. zone between η phases can be restrained because surrounding Mg and Zn atoms are easy to be captured by those larger η phases, and therefore, such G.P. zone can be wrapped by more Al atoms, which explains why a certain quantity of G.P. zones can still exist after sufficient aging.

## Abbreviations

3D: Three-dimensional
APT: Atom probe tomography
EDS: Energy dispersive spectroscopy
HRTEM: High-resolution transmission electron microscopy
IVAS: Imago Visualization and Analysis Software
MPts: Matrix precipitates
OA: T73 in over aging
PAI: Peak aging I
PAII: peak aging II
*R*_eq_: Equivalent radius
SAED: Selected area electron diffraction
TEM: Transmission electron microscopy
UA: Under aging
